# Correlation Between Hemoglobin A1c (HbA1c) and High-Sensitivity C-Reactive Protein (hs-CRP) in Myocardial Infarction Patients and Their Six-Month Mortality Follow-Up

**DOI:** 10.7759/cureus.67070

**Published:** 2024-08-17

**Authors:** Keesari Sai Sandeep Reddy, Priyadarshini Varadaraj, Gunasekaran Nallusamy, Subbiah SenthilNathan

**Affiliations:** 1 Internal Medicine, Saveetha Medical College and Hospital, Saveetha Institute of Medical and Technical Sciences (SIMATS) Saveetha University, Chennai, IND

**Keywords:** type 2 diabetes mellitus, hba1c, hs-crp, myocardial infarction, acute coronary syndrome

## Abstract

Background

Acute coronary syndrome (ACS), encompassing unstable angina (UA), non-ST-elevation myocardial infarction (NSTEMI), and ST-elevation myocardial infarction (STEMI), poses significant global health challenges because of its associated high mortality and morbidity rates. Vascular inflammation plays a crucial role in the pathogenesis of atherosclerosis, and it is often assessed using biomarkers such as high-sensitivity C-reactive protein (hs-CRP). Hyperglycemia, common in myocardial infarction patients, is linked to increased complications and mortality, with glycosylated hemoglobin A1c (HbA1c) serving as a key indicator of long-term glycemic control.

Objective

This study investigates the correlation between hs-CRP and HbA1c levels in patients with acute myocardial infarction (AMI) and type 2 diabetes mellitus (T2DM) and evaluates their impact on six-month mortality outcomes.

Methods

A prospective observational study was conducted with 80 patients diagnosed with AMI. Data collection included demographic information, medical history, clinical assessments, laboratory investigations (including hs-CRP and HbA1c levels), and imaging studies. Patients received standard treatment and were followed up for six months. Statistical analyses were performed to examine the relationships between hs-CRP, HbA1c, and clinical outcomes.

Results

Higher HbA1c levels at admission were significantly correlated with elevated hs-CRP levels (p < 0.05). Both biomarkers showed a reduction at six months, correlating with improved glycemic control and reduced inflammation. Each unit increase in HbA1c was associated with a 21% increase in the hazard of mortality, and, similarly, each unit increase in hs-CRP was associated with a 17% increase in the hazard of mortality. The positive correlation between HbA1c and hs-CRP suggests that HbA1c can serve as an independent marker for predicting mortality in this patient population.

Conclusion

The study demonstrates a significant correlation between hs-CRP and HbA1c levels in patients with AMI and T2DM, with both biomarkers serving as strong predictors of six-month mortality. HbA1c, because of its positive correlation with hs-CRP, could be used as an independent marker for assessing the risk of adverse outcomes in these patients. These findings highlight the importance of managing both glycemic control and inflammation in diabetic patients with ACSs.

## Introduction

Acute coronary syndrome (ACS) is a term that encompasses a range of thrombotic coronary artery diseases, including unstable angina (UA), non-ST-elevation myocardial infarction (NSTEMI), and ST-elevation myocardial infarction (STEMI). These conditions are major global health issues, significantly contributing to hospitalizations and posing considerable challenges because of their associated complications, which often lead to high rates of mortality and morbidity [1]. The diagnosis of ACS typically relies on the presence of characteristic symptoms, electrocardiographic (ECG) changes, variations in cardiac biomarkers, and evidence of recent myocardial damage [2].

Vascular inflammation is known to precede clinical cardiovascular syndromes and plays a crucial role in the pathogenesis of atherosclerosis, contributing to the formation and destabilization of atherosclerotic plaques [3]. However, the direct mechanisms underlying these inflammatory processes are not fully understood, and practical, noninvasive methods for assessing coronary vessel inflammation are limited. Consequently, various serum inflammatory biomarkers, such as serum amyloid A, fibrinogen, interleukin-6 (IL-6), apolipoprotein A, and highly sensitive C-reactive protein (hs-CRP), have been investigated as potential tools for predicting inflammatory activity in ACS [4].

Hs-CRP has emerged as a valuable marker for assessing acute coronary events and the effectiveness of therapeutic interventions. It has been widely studied and is recognized not only as an indicator of generalized inflammation but also as an active participant in atherogenesis and plaque disruption [5]. Furthermore, hyperglycemia, often observed in myocardial infarction (MI) patients, is directly associated with increased morbidity and mortality, even among those without a prior diagnosis of diabetes [6]. Glycosylated hemoglobin A1c (HbA1c), which reflects average blood glucose levels over the preceding three months, is a key biomarker for assessing glycemic control and predicting complications [7].

This study aims to investigate the correlation between hs-CRP and HbA1c levels in patients with acute myocardial infarction (AMI) and T2DM, focusing on their impact on six-month mortality outcomes.

## Materials and methods

This study was designed as a prospective observational study aimed at investigating the correlation between hs-CRP and HbA1c levels in patients with AMI and T2DM, focusing on their impact on six-month mortality.

The study included 80 patients diagnosed with AMI. This was conducted over ten months. A convenience sampling method was employed to enroll participants. All eligible patients admitted with STEMI during the study period were included.

Participants were included if they were newly diagnosed with STEMI, were over 18 years of age, had a known history of T2DM, or had an HbA1c level greater than 6.5% on admission. Patients who had not previously received any antiplatelet or statin therapy were considered eligible.

Exclusion criteria were established coronary artery disease, ongoing statin or anti-inflammatory drug therapy known to reduce CRP levels, hemolytic anemias, total leukocyte counts greater than 11,000 or less than 4,000, recent onset of fever within the past week, chronic kidney disease, chronic liver disease, a history of myocardial infarction or heart failure, and malignancy.

Data were collected from all subjects using a structured data collection form. This form included basic demographic information such as age, sex, occupation, and history of substance abuse, including smoking and alcohol consumption. Medical history was documented, noting the presence of concomitant diseases and previous medical treatments. Clinical assessments involved the confirmation of STEMI via ECG and documentation of its characteristics. Laboratory investigations included cardiac biomarkers, specifically troponin T and CPK-MB, to confirm the diagnosis. Blood samples were taken to measure HbA1c and hs-CRP levels.

Treatment for patients arriving within the stipulated time for percutaneous coronary intervention (PCI) included angiography and stenting if financially feasible. Patients who arrived beyond the stipulated time or those who declined PCI for economic reasons received thrombolytic therapy with streptokinase, alteplase, or tenecteplase. The standard therapy for all patients included dual antiplatelets (aspirin and clopidogrel), high-dose statins, angiotensin-converting enzyme (ACE) inhibitors, beta-blockers, and diuretics. Informed consent was obtained from all patients or their relatives prior to angiography or thrombolysis.

Patients were monitored until discharge for any complications. Following discharge, patients were followed up every two months to document any mortality. At the end of six months, surviving patients underwent repeat measurements of HbA1c and hs-CRP levels.

Data were analyzed using Statistical Product and Service Solutions (SPSS; IBM SPSS Statistics for Windows, Armonk, NY). Descriptive statistics were used to summarize demographic and clinical characteristics. Pearson correlation coefficients were calculated to assess the relationship between HbA1c and hs-CRP levels at admission and their changes over six months. Multivariable Cox proportional hazards regression models were employed to identify independent predictors of six-month mortality, adjusting for potential confounders such as age, sex, treatment modality (thrombolysis vs. PCI), presence of comorbid conditions (e.g., hypertension, dyslipidemia, and previous history of cardiovascular events), smoking status, and alcohol consumption. By adjusting for these variables, we aimed to minimize the impact of these confounders on the association between the main predictors (HbA1c and hs-CRP) and the outcomes. Statistical significance was set at a P value of less than 0.05.

Ethical considerations were thoroughly addressed. The study protocol was reviewed and approved by the institutional ethics committee. All participants provided informed consent before enrollment in the study. Patient confidentiality and data privacy were strictly maintained throughout the study.

## Results

Table 1 shows the distribution of hs-CRP levels and HbA1c levels at admission and six months later. There was a significant decrease in hs-CRP levels over the six-month period, although no patients reached normal levels (< 1 mg/L). There was a significant reduction in HbA1c levels over six months, with the highest percentage of patients moving from HbA1c > 11% to lower categories.

**Table 1:** hs-CRP levels and HbA1c levels on admission vs at six months

hs-CRP Levels on Admission vs at Six Months				
hs-CRP Level (mg/L)	Frequency on Admission	Percentage on Admission (%)	Frequency at Six Months	Percentage at Six Months (%)
<1	0	0	0	0
1-3	2	2.5	18	26.5
>3	78	97.5	50	73.5
HbA1c Levels on Admission vs at Six Months				
HbA1c Level (%)	Frequency on Admission	Percentage on Admission (%)	Frequency at Six Months	Percentage at Six Months (%)
<7	6	7.5	16	23.5
7-8.9	26	32.5	38	55.8
9-10.9	20	25	14	20.5
>11	28	35	0	0

Table 2 depicts the relationship between HbA1c levels and hs-CRP levels at admission and at six months. There is a clear trend of higher hs-CRP levels with higher HbA1c levels, indicating a connection between poor glycemic control and higher inflammation during admission. After six months, there is a reduction in both HbA1c and hs-CRP levels across the board, with lower HbA1c levels corresponding to lower hs-CRP levels.

**Table 2:** HbA1c vs hs-CRP on admission and at six months

HbA1c vs hs-CRP on Admission		
HbA1c Level (%)	hs-CRP Level (mean, mg/L)	Standard Deviation
<7	5.117	3.0505
7-8.9	5.819	1.0427
9-10.9	6.87	1.1707
>11	9.779	1.6976
Mean	7.415	2.3542
HbA1c vs hs-CRP at Six Months		
HbA1c Level (%)	hs-CRP Level (mean, mg/L)	Standard Deviation
<7	3.221	1.1102
7-8.9	3.564	0.7394
9-10.9	4.223	0.9218
>11	-	-
Mean	3.665	0.739

Pearson correlation coefficients were calculated to assess the connection between HbA1c and hs-CRP levels at both admission and at six months. A significant positive connection was found between HbA1c and hs-CRP levels at admission (correlation coefficient(r) = 0.81, p < 0.001) and at six months (correlation coefficient (r) = 0.75, p < 0.001). These results indicate that higher HbA1c levels are associated with higher hs-CRP levels, suggesting a link between poor glycemic control and increased inflammation.

Table 3 illustrates the mortality rates at six months based on HbA1c levels and hs-CRP levels at admission. The highest mortality rate is observed in patients with HbA1c > 11%, indicating that higher HbA1c levels at admission are associated with increased mortality. All patients who died had hs-CRP levels > 3 mg/L, suggesting that high hs-CRP levels at admission are associated with increased mortality.

**Table 3:** Mortality of patients at six months with HbA1c and hs-CRP on admission

Mortality of Patients at Six Months with HbA1c on Admission			
HbA1c Level on admission (%)	Deaths	Alive	Total
<7	0	6	6
7-8.9	2	24	26
9-10.9	0	20	20
>11	10	18	28
Mortality of Patients at Six Months with hs-CRP on Admission			
hs-CRP Level (mg/L)	Deaths	Alive	Total
<1	0	0	0
1-3	0	2	2
>3	12	66	78

Cox proportional hazards regression was performed to identify independent predictors of six-month mortality. The hazard ratios (HR) for HbA1c and hs-CRP levels were calculated, adjusting for potential confounders. The results are mentioned in Table 4. Higher HbA1c and hs-CRP levels at admission were identified as significant independent predictors of increased six-month mortality. Each unit increase in HbA1c was associated with a 21% increase in the hazard of mortality (HR = 1.21, 95% confidence interval (CI): 1.08-1.36, p < 0.001), while each unit increase in hs-CRP was associated with a 17% increase in the hazard of mortality (HR = 1.17, 95% CI: 1.04-1.31, p = 0.009).

**Table 4:** Cox proportional hazards regression results

Variable	Hazard Ratio (HR)	95% Confidence Interval (CI)	P value
HbA1c (Admission)	1.21	1.08-1.36	<0.001
hs-CRP (Admission)	1.17	1.04-1.31	0.009

## Discussion

This study aimed to explore the correlation between hs-CRP and HbA1c levels in patients with AMI and T2DM, focusing on their impact on six-month mortality outcomes. The findings indicate a significant relationship between poor glycemic control, as reflected by elevated HbA1c levels, and increased inflammatory activity, as indicated by hs-CRP levels, both at admission and after six months.

The results show that higher HbA1c levels are associated with elevated hs-CRP levels at admission. This aligns with previous studies that have demonstrated a link between hyperglycemia and increased systemic inflammation in patients with cardiovascular diseases [8]. Hyperglycemia is known to promote the formation of advanced glycation end-products (AGEs), which can induce oxidative stress and inflammatory responses, contributing to the pathogenesis of atherosclerosis and destabilization of atherosclerotic plaques [9].

At six months, both HbA1c and hs-CRP levels decreased significantly across the board. This suggests that improved glycemic control and standard medical therapy such as dual antiplatelet therapy (aspirin and clopidogrel), high-dose statins (e.g., atorvastatin), ACE inhibitors, beta-blockers, and lifestyle modifications contribute to reducing systemic inflammation. The reduction in hs-CRP levels is particularly noteworthy, as it underscores the role of inflammation in the progression of coronary artery disease and the potential benefit of targeting inflammatory pathways as part of comprehensive cardiovascular risk management [10].

While treatment modality and clinical stability are important considerations, the significant associations observed with hs-CRP and HbA1c levels suggest that these biomarkers are key indicators of long-term mortality risk, irrespective of the immediate treatment received.

The relationship between high hs-CRP levels and increased six-month mortality highlights the prognostic value of hs-CRP in patients with AMI and T2DM. Patients with hs-CRP levels > 3 mg/L at admission had a significantly higher mortality rate compared to those with lower levels. This finding is consistent with existing literature that recognizes hs-CRP as a strong predictor of adverse cardiovascular outcomes and mortality [11]. High hs-CRP levels reflect heightened inflammatory activity, which can exacerbate myocardial damage and contribute to complications such as heart failure and recurrent ischemic events [12].

Importantly, this study found that HbA1c levels >11% at admission were associated with the highest mortality rates, emphasizing the critical importance of glycemic control in reducing mortality risk in diabetic patients with AMI. Given the strong correlation between HbA1c and hs-CRP levels, our findings suggest that HbA1c could serve as an independent marker of morbidity and mortality in this patient population. Poor glycemic control is associated with various adverse effects, including endothelial dysfunction, impaired fibrinolysis, and increased platelet aggregation, all of which can worsen cardiovascular outcomes [13]. Therefore, achieving optimal glycemic control should be a priority in the management of diabetic patients with ACSs [14].

Our findings support the integration of HbA1c measurements into routine clinical practice for risk stratification and management of patients with AMI and T2DM. Monitoring HbA1c levels can provide valuable insights into the glycemic status of patients and guide therapeutic decisions aimed at reducing hyperglycemia and its associated risks. Given the proven correlation between HbA1c and hs-CRP, HbA1c could be a reliable marker for identifying patients at higher risk of adverse outcomes, thereby enabling more targeted and effective interventions [15].

Strengths and limitations of the study

Strengths

Prospective design: The prospective nature of this study allowed for the systematic collection of data, reducing the risk of recall bias and improving the reliability of our findings.

Use of regression models: We utilized regression models to account for known confounders, such as age, sex, baseline HbA1c levels, and treatment modality, which strengthens the validity of our results.

Focus on glycemic control and inflammation: This study contributes to the understanding of the relationship between glycemic control (as measured by HbA1c) and systemic inflammation (as measured by hs-CRP) in patients with AMI and T2DM.

Limitations

Unknown confounders: Despite our efforts to adjust for known confounders, the possibility of unknown confounders affecting the study outcomes cannot be excluded. Factors such as patient adherence to prescribed therapies and variations in post-discharge care might have influenced the results.

Treatment modality: The study included patients treated with both thrombolysis and PCI, and we acknowledge that patients treated with PCI generally experience better long-term outcomes compared to those treated with thrombolytics. Moreover, among thrombolytics, tenecteplase has been shown to have superior efficacy compared to streptokinase, which may have affected the overall outcomes. The differences in treatment efficacy could have introduced variability in the reduction of HbA1c and hs-CRP levels, potentially impacting our findings.

Sample size: While the sample size of 80 patients provided sufficient data for analysis, larger studies are needed to validate our findings and to explore the impact of different treatment modalities on long-term outcomes more comprehensively.

Follow-up duration: The follow-up period of six months, while sufficient to observe significant changes in HbA1c and hs-CRP levels, may not capture the full extent of long-term cardiovascular outcomes and requires further study.

## Conclusions

In conclusion, our study demonstrates a significant correlation between hs-CRP and HbA1c levels in patients with AMI and T2DM, with both biomarkers being strong predictors of six-month morbidity and mortality. The positive correlation between HbA1c and hs-CRP suggests that HbA1c could serve as an independent marker of morbidity and mortality in this patient population. These findings highlight the importance of addressing both inflammation and glycemic control in the management of diabetic patients with ACSs. Future research should focus on larger, multicenter studies and explore the potential benefits of combined anti-inflammatory and glucose-lowering therapies in this high-risk population.
